# Implementation of recommended trauma system criteria in south-eastern Norway: a cross-sectional hospital survey

**DOI:** 10.1186/1757-7241-20-5

**Published:** 2012-01-26

**Authors:** Thomas Kristiansen, Kjetil G Ringdal, Tarjei Skotheimsvik, Halvor K Salthammer, Christine Gaarder, Pål A Næss, Hans M Lossius

**Affiliations:** 1 Department of Research, Norwegian Air Ambulance Foundation, Drøbak, Norway; 2 Department of Anaesthesiology, Vestre Viken Hospital Trust, Kongsberg, Norway; 3 Institute of Clinical Medicine, Faculty of Medicine, University of Oslo, Oslo, Norway; 4 Faculty of Medicine, University of Oslo, Oslo, Norway; 5 Department of Traumatology, Emergency Division, Oslo University Hospital, Oslo, Norway; 6 Department of Surgical Sciences, University of Bergen, Bergen, Norway

## Abstract

**Background:**

Formalized trauma systems have shown beneficial effects on patient survival and have harvested great recognition among health care professionals. In spite of this, the implementation of trauma systems is challenging and often met with resistance.

Recommendations for a national trauma system in Norway were published in 2007. We wanted to assess the level of implementation of these recommendations.

**Methods:**

A survey of all acute care hospitals that receive severely injured patients in the south-eastern health region of Norway was conducted. A structured questionnaire based on the 2007 national recommendations was used in a telephone interview of hospital trauma personnel between January 17 and 21, 2011. Seventeen trauma system criteria were identified from the recommendations.

**Results:**

Nineteen hospitals were included in the study and these received more than 2000 trauma patients annually via their trauma teams. Out of the 17 criteria that had been identified, the hospitals fulfilled a median of 12 criteria. Neither the size of the hospitals nor the distance between the hospitals and the regional trauma centre affected the level of trauma resources available. The hospitals scored lowest on the criteria for transfer of patients to higher level of care and on the training requirements for members of the trauma teams.

**Conclusion:**

Our study identifies a major shortcoming in the efforts of regionalizing trauma in our region. The findings indicate that training of personnel and protocols for inter-hospital transfer are the major deficiencies from the national trauma system recommendations. Resources for training of personnel partaking in trauma teams and development of inter-hospital transfer agreements should receive immediate attention.

## Background

Formalized trauma systems were described more than three decades ago [1]. Supported by an increasing amount of empirical evidence, the benefit of trauma systems have been widely accepted among trauma care providers [2-5]. In spite of this, relatively few regions internationally have fully implemented the trauma system concepts. Factors that make trauma system implementation challenging, like financial costs, lack of political will, and resistance against centralizing health care services, have been identified [6-9]. Several measures that facilitate implementation have also been proposed: research documenting the need for change, continuous quality improvement and a broad based organizational leadership [9-12]. In several regions the implementation of a trauma system has been traced back to a dramatic or tragic event and the importance of trauma-enthusiastic professionals has also been highlighted [9,11-15].

In Europe, trauma systems are the exception rather than the rule [16]. Germany has documented reduction in mortality after regionalization of trauma care [5,17]; in the UK, the recently developed London trauma system is considered the first step towards a national trauma system implementation in England [7].

In the Scandinavian countries, there are many hospitals receiving relatively few trauma patients [18]. Elements of the trauma system principles have been implemented in certain regions [19,20], however, there are still no formalized regional trauma systems [18].

Recommendations for developing a national trauma systems in Norway were published in September 2007 [21]. The recommendations included criteria for trauma resources for hospitals that receive trauma patients. The aim of this study was to assess the level of implementation of the trauma system recommendations in acute care hospitals in the south-eastern health region of Norway.

## Methods

**Figure 1:**
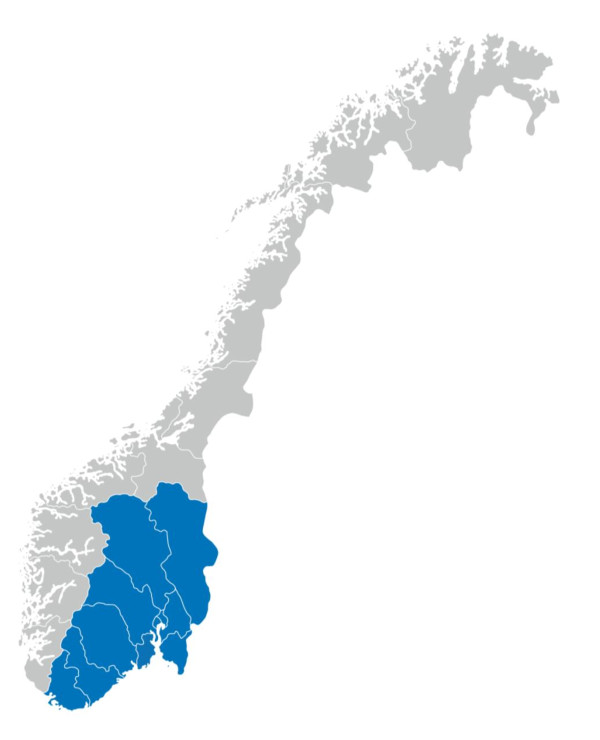
**Map of Norway showing the jurisdiction of the South-Eastern Norway Regional Health Authority**.

The South-Eastern Norway Regional Health Authority covers an area of 111.000 km^2 ^with 2.7 million inhabitants (Figure 1). The region's trauma services consist of 19 acute care hospitals located outside Oslo and one trauma referral centre situated in Oslo. There are three additional hospitals in Oslo with emergency surgical services. However, all suspected major trauma patients in Oslo are triaged according to formalized criteria directly to the trauma centre at Oslo University Hospital - Ullevål (OUH-U). The recommendations for a Norwegian trauma system proposed two levels of trauma receiving hospitals: acute care hospitals and trauma centres. The requirements and functions of acute care hospitals, are similar to those described for Level 3 and Level 4 trauma centres by the American College of Surgeons [22]. The trauma centres should fulfil requirements similar to Level 1 and Level 2 trauma centres by the American definitions. However, there is currently no accreditation system that certifies Norwegian hospitals. The OUH-U trauma service has been described previously [23,24]. In this study we therefore included all hospitals with emergency surgical services in the south-eastern health region located outside of Oslo.

### Data collection and variable definitions

The data were collected by telephone survey using a structured questionnaire (Additional File 1). The data collection took place between January 17 and 21, 2011. All hospitals in the south-eastern health region of Norway were contacted. The respondents were the hospital trauma coordinator, the consultant in charge of trauma or the head of the emergency department.

Seventeen specific criteria for acute care hospitals were identified from the Norwegian trauma system recommendations (Table 1). The respondents were asked to describe their hospital's status on the questionnaire items at the time of the interview and to report the minimum level of resources available at all times.

**Table 1:** Seventeen trauma system criteria and definitions identified from the recommendations for a national trauma system in Norway -reference 21.

**Item no**.	Criteria	*Definitions *
1	Defined TT	*-a defined multidisciplinary group of personnel receives trauma patients*

2	TT activation criteria	*-predefined and written criteria activates the TT*

3	TT activation < 15 min	*-the time to assemble the TT is within 15 minutes*

4	TT available 24 hrs	*-the TT is accessible around the clock*

5	TT training	*-there are regular training sessions for the TT with a minimum frequency of two times per year. TT training is based on the principles described by the BEST foundation (see references 25, 30 and 31) *

6	ED < 15 min	*-the emergency room is ready within 15 minutes*

7	OR < 15 min	*-the operating theatre is ready within 15 minutes*

8	CXR < 15 min	*-a chest x-ray is taken and made accessible to the TT within 15 min*

9	Trauma Protocol	*-there is a written and updated trauma protocol describing the management of major trauma*

10	Trauma Checklist	*-a checklist is used for documenting and guiding the management of the trauma patient in the ED*

11	Transfer Criteria	*-there are written criteria for transfer of patients to higher level of care*

12	Trauma Registry	*-the hospital records data of trauma patients in a dedicated registry*

13	Trauma Meetings	*-the hospital conducts regular morbidity and mortality meetings. The meetings are multidisciplinary audits where management of the hospital's trauma patients are discussed*. *The minimum frequency is two times per year*.

14	Anaesthesiologist ATLS- course	*-the trauma team senior anaesthesiologist is required to have attended the ATLS- course*

15	Surgeon ATLS- course	*-the trauma team senior surgeon is required to have attended the ATLS- course*.

16	Haemostatic surgery course	*-the trauma team senior surgeon is required to have attended DSTC or equivalent emergency haemostatic surgery course*

17	Trauma nursing course	*-minimum one of the trauma team nurses is required to have attended TNCC course or equivalent*.

Abbreviations denote: TT: trauma team; BEST: Better & Systematic Trauma Care; ED: emergency department; OR: operating room; CXR: chest x-ray; ATLS: advanced trauma life support; DSTC: definitive surgical trauma care course; TNCC: trauma nursing core course

Some criteria were grouped into related categories. Criteria 1-5, regarding trauma teams, were grouped as one category as these are the criteria for trauma teams in acute hospitals in the 2007 recommendations. Further, these criteria, including regular team training, reflect the BEST foundation initiatives for trauma teams in Norwegian hospitals [25]. Criteria 6-8 reflect some of the high-cost recommendation for preparedness, while the criteria 9 and 10 reflect the institutional level of medical direction. Criteria 14 to 17, categorized as training of personnel, represent training requirements for individuals of different professions. Unlike the trauma team training criteria, these are relatively high-cost criteria with standardized courses held outside the workplace of the participants.

For criteria regarding training of personnel and regarding the availability of senior staff, the respondents were asked to report on the trauma team leaders or the most senior personnel present in the trauma team, or if there were no defined teams, the personnel in charge of the management of trauma patients.

Data on the population in the primary catchment area, defined as the number of inhabitants within the area from where each hospital receives trauma patients, were primarily collected from the respondents. For the three hospitals where the respondents were unable to provide these estimates, this information was gathered from the Norwegian Directorate of Health. For comparison purposes, the hospitals were divided into "small": primary population ≤ 85.000, and "large": primary population > 85.000. The distance to the trauma centre is the number of kilometres, by ground transport, between each hospital and OUH-U. For comparisons, the hospitals were divided into "short distance": ≤ 122 km, and "long distance": > 122 km.

### Statistical analysis

Results are presented as medians with interquartile ranges (IQR). Proportions are presented as number of hospitals and the denominator is 19 hospitals, unless otherwise specified. Due to the low number of study objects (*n *= 19 hospitals), statistical tests for categorical variables were not considered appropriate. The association between the distance to OUH-U vs. the number of fulfilled criteria, and the primary population vs. the number of fulfilled criteria, was estimated by univariate linear regression. All tests were two-tailed and statistical significance was assumed for *p *< 0.05. SPSS v.19.0 (IBM Company, Chicago, IL) was used for analysis.

The Regional Committee for Medical and Health Research Ethics was informed and stated that the study did not require formal ethical approval (Ref.no: 2010/2665-1).

## Results

There were 19 hospitals included in the study, all of which received severely injured patients from within their primary uptake area. The median population within the hospitals' primary uptake areas was 85.000 (IQR 40.000-180.000) and the median distance from the hospitals to the trauma centre was 122 km (IQR 82-191).

Data from trauma admissions were systematically gathered in a trauma registry in 11 out of the 19 hospitals. Of these 11 hospitals, five had exact data on the number of trauma team activations (TTA) per year and five reported estimated numbers. Of the remaining eight hospitals without trauma registries, two had exact numbers of annual TTA and five reported estimated numbers. Two hospitals were not able to report on annual TTA. The sum of the annual TTA for the 17 hospitals that reported either estimated or exact numbers was 2399 and the median number of annual TTA for these hospitals was 110 (IQR 57-226).

Of the 17 defined trauma system criteria for this study, the median number of fulfilled criteria across the hospitals was 12 (IQR 11-16), and only one hospital fulfilled all the criteria (Table 1). There was no association between the size of the hospitals' primary uptake area and the number of criteria fulfilled (R^2^: 0.027; P = 0.5), neither was there any association between the distance from the hospitals to the trauma centre and number of criteria fulfilled (R^2^: 0.004; P = 0.8).

### Trauma Teams

**Figure 2:**
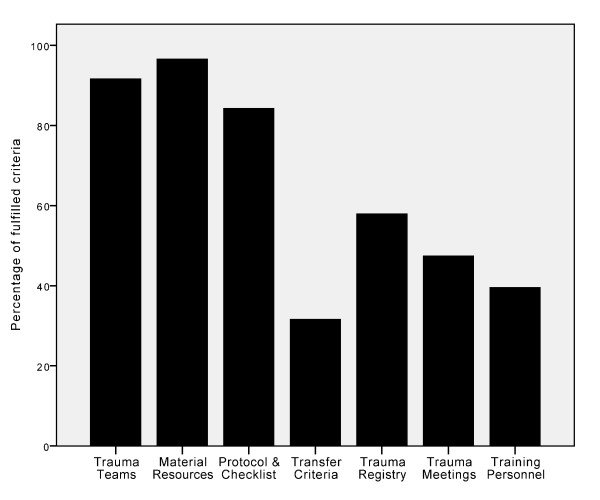
**Percentage of fulfilled criteria by all 19 hospitals stratified by different criteria categories**.

The criteria regarding trauma teams (items 1 to 5, Table 1) were generally well covered in the hospitals. Of the 19 hospitals, 15 fulfilled all the five criteria regarding trauma teams and in total, 92% of these criteria were fulfilled (Figure 2). Seventeen of the hospitals had regular trauma team training and the average frequency of training was once every nine weeks.

### Material Resources

One hospital reported that it would take more than 15 minutes to have an operating room ready and one hospital was not able to have a chest X-ray available within 15 minutes. With these two exceptions, the criteria regarding material resources (items 6 to 8, Table 1) were fulfilled by all hospitals (Figure 2).

### Protocols and Checklists

The majority of the hospitals (16 out of 19) had updated written trauma management protocols and used a trauma checklist to document and guide the management of patients in the emergency department (items 9 and 10, Table 1; Figure 2).

### Inter-hospital Transfer

All the hospitals in the region transferred patients in need of tertiary trauma services to the regional trauma centre. However, only six of the 19 hospitals had implemented predefined written criteria for inter-hospital transfer (item 11, Table 1; Figure 2).

### Trauma Meetings

Less than half of the hospitals reported having regular trauma audit meetings (item 13, Table 1; Figure 2). For the larger hospitals, six out of nine hospitals had regular trauma meetings, while for the smaller hospitals this number was three out of ten.

### Training of Personnel

In general, hospitals had low scores on implementation of the recommended criteria for training of the trauma team personnel (Figure 2). Out of the four defined criteria (items 14 to 17, Table 1), the median number of fulfilled requirements per hospital was two (IQR 1-4). Only two hospitals met all the criteria, and four hospitals had none of the criteria fulfilled. Less than one third of the hospitals required haemostatic emergency surgery courses and trauma nursing courses, for surgeons and nurses, respectively. These were thus the least frequently fulfilled criteria for training of personnel. ATLS-course attendance for surgeons was a requirement for ten out of 19 hospitals, while this number was eight out of 19 hospitals with respect to the anaesthesiologists.

### Availability of Senior Staff

**Figure 3:**
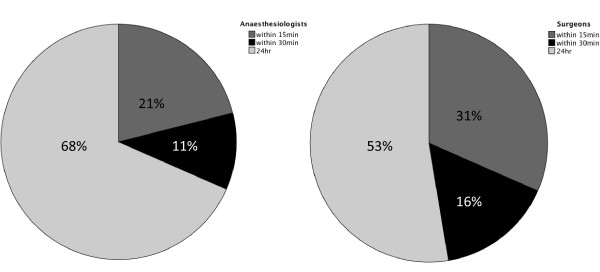
**Pie chart with percentages showing availability of senior surgeons and anaesthesiologists stratified according to 24-hour availability, within 15 minutes and within 30 minutes**.

The anaesthesiologists were more immediately accessible during out of office hours than the surgeons. See Figure 3 for the two professions' distribution on the different categories of availability.

## Discussion

Our survey of 19 acute care hospitals in the southeast of Norway indicate that many of the hospital components for trauma systems are in place, but that the hospitals score poorly on the criteria for training of the trauma team members. Another major deficit found in this study is that few hospitals had implemented criteria for inter-hospital transfer of patients to higher level of care; this finding is identical to that of a national U.S. survey assessing trauma system implementation in 1995 [8]. Inter-hospital transfer is a key component that reflects the level of coordination of a trauma system [8,26,27]. As such, this study identifies a major shortcoming in the efforts of regionalizing trauma in our region.

The vast majority of trauma team leaders in Norway are surgeons. Similar to the majority of European countries, trauma surgery is not a separate specialty in Norway and most Norwegian hospitals admit few severely injured patients [18,28]. It is therefore concerning that so few surgeons have attended the recommended surgery courses and that a considerable part of the senior personnel were less than readily available during out of office hours.

This study included all hospitals that receive severely injured patients in the south-eastern health region, apart from the regional trauma centre. As these hospitals receive more than 2000 patients per year via their trauma teams, the future quality of care in the acute care hospitals in the region is important and will affect a large number of persons in the years to come.

Identified facilitators of trauma system implementation include documenting a need for change and continuous quality improvement [9-12]. Studies that assess the current level of trauma resources and the epidemiology of trauma within a region, are further proposed as initial steps that enable appropriate direction of trauma system development [10,29]. The regional trauma centre, OUH-U, maintains a trauma registry that has enabled several benchmarking studies [23,24]. This study shows that eleven of the non-trauma centre hospitals in our region also maintain trauma registries. However, individual institutional registries do not necessarily gauge the need for trauma system implementation; the performance of the trauma system constitutes the pre-hospital service providers', the non-trauma centre and trauma centre hospitals' individual performance and their coordinated interaction. Regional benchmarking thus requires population-based data capture in regional or national trauma registries. The Norwegian trauma system recommendations advocate a national trauma registry [21]. This is still not implemented in Norway or any other of the Scandinavian countries [18]. There is a scarcity of studies that document a need for change at a system level in Scandinavia and this may be partly due to this lack of data registries.

The implementation of the trauma system recommendations until now can be ascribed to local initiatives with the facilitation of trauma enthusiasts such as the BEST network [30] and, in our region, the networking led by the regional trauma centre, OUH-U. The majority of hospitals in Norway have participated in the BEST foundations effort to improve trauma care through trauma courses [31]. The high coverage of the trauma team criteria, the material resources criteria and the use of checklists and trauma protocols in the ED, are in accordance with the emphasis in the BEST courses. Furthermore, 14 out of the 16 trauma checklists in use in the study hospitals were those developed by the BEST foundation.

Experiences from other regions indicate that "elite consensus" may be insufficient to implement a regional trauma system. The development of a trauma system is a process of allocating trauma resources and matters of financial costs and political decision making inevitably affect progress. A comprehensive assessment of the funding required to make the desired changes were among the recommended key initiatives when developing the trauma system in the state of Minnesota [32]. In a large US survey [9], regions that successfully implemented trauma systems benefitted from a strong and inclusive organizational leadership with members from both trauma centre and non-trauma centre hospitals, pre-hospital service providers, as well as political and community stakeholders. Anchoring the trauma system within a comprehensive legislation will also facilitate implementation by securing adherence to policies and may facilitate securing financial resources [10].

In December 2010 the board of the South-Eastern Norway Regional Health Authority voted in favour of a proposal to support the implementation of the 2007 trauma system recommendations. The proposal included concrete criteria based on the 2007 recommendations and a time schedule with dates for implementing the different criteria. The completion of the proposed recommendations is in June 2012. This board decision gives reason for optimism regarding trauma system implementation in our region as it includes many of the facilitating factors identified from successful implementation elsewhere. Furthermore, the proposal states that The South-Eastern Norway Regional Health Authority should assist with implementation in the three other health regions in Norway, and thus facilitate national trauma system implementation. The formal process leading to the board proposal has been initiated and led by the Department of Traumatology at OUH-U. This shows the importance of the trauma community's communication and education of key stakeholders and has also been emphasised in previous studies [11,12].

Trauma enthusiasts have already shown willingness and ability to improve trauma care in Norway. Further development should follow the example of regions that have successfully implemented trauma systems and will require organizing regional and national trauma registries, detailed analyses of the resources required to implement and maintain a trauma system, defining national and regional organizational leadership, and finally, working towards legislative changes that secure adherence to the principles of improved trauma care.

## Limitations

This study was conducted by telephone interview where respondents were asked to report according to predefined categories (Additional File 1). This method of data collection may foster communication difficulties such as unforeseen ambiguity in questions [33]. A pilot test of the questionnaire prior to the study could have reduced some of the ambiguity respondents experienced and could have reduced the need for clarifications by the interviewers during the interview.

The resources available in any hospital may fluctuate by chance according to which personnel is on call, and systematically, by more junior personnel being present during less favourable hours and days. The responders were therefore asked to report only those resources that would be available at all times. However, if one assumes that respondents would like to project a high level of trauma competence for their respective institutions, our method of data collection may be prone to a response bias. It may therefore be more likely that the study overestimates, rather than underestimates, the level of trauma resources in the region.

The study aimed to assess the concrete criteria from the trauma system recommendations published in 2007. However, this publication is not complete in all aspects regarding trauma systems. Availability of certain resources such as computed tomography scanners was not defined in the trauma system recommendations and was therefore not included in the study questionnaire.

## Conclusion

The recommendations for a national trauma system were published in 2007. Our survey of all acute care hospitals in the southeast health region of Norway identifies a major shortcoming in the efforts of regionalizing trauma in this region. The findings indicate that training of personnel and protocols for inter-hospital transfer are the major deficiencies from the recommendations. Further strategies should include benchmarking of regional performance through population-based trauma registries and defining organizational responsibility for regional and national implementation.

## List of abbreviations

OUH-U: Oslo University Hospital Ullevål; IQR: interquartile range; TTA: trauma team activation; ATLS: advanced trauma life support; BEST: better and systematic trauma care; ED: emergency department; EMS: emergency medical service.

## Competing interests

The authors declare that they have no competing interests.

## Authors' contributions

TK, KGR and HML had the original idea for the study. The study protocol was discussed, written and edited by all authors. The questionnaire was developed by TK, KGR, TS and HS. The telephone interviews were done by TK, TS and HS. TK developed the database and analysed the data. The manuscript was drafted by TK and all authors contributed to the editing and accepted the final version of the article.

## Supplementary Material

Additional file 1 **Structured Questionnaire**. Click here for file
