# Supplementary material for: Implementation of recommended trauma system criteria in south-eastern Norway: a cross-sectional hospital survey
Source: Scand J Trauma Resusc Emerg Med. 2012 Jan 26;20:5. doi: 10.1186/1757-7241-20-5 (PMC3285082; doi:10.1186/1757-7241-20-5)
Supplement: Additional file 1 — Structured Questionnaire. [file 1757-7241-20-5-S1.DOC]

**Structured Questionnaire**

Telephone interviews 17. - 21. January 2011

*Respondents:*

-hospital trauma coordinator or the BEST network contact person

-consultant in charge of trauma

-head of the emergency department

***Initial questions***

1. Does the hospital perform acute surgical services?

▭Yes

▭No

2. Does the hospital accept potentially seriously injured patients?

▭Yes

▭No

If yes, proceed to the question below:

1. Does the hospital have a predefined trauma team?

▭Yes

▭No

2. Does the hospital have predefined criteria for activation of the trauma team?

▭Yes

▭No

3. Does it take less than 15 min. to activate the trauma team?

▭Yes

▭No

4. Can the trauma team be mobilized around the clock?

▭Yes

▭No

5. Does the hospital conduct regular trauma team training sessions?

▭Yes (minimum twice per year)

▭No

6. How often does the hospital implement team training?

___________ (Exact, what year)

___________ (Estimated)

7. Does it take less than 15 min. to prepare the emergency room?

▭Yes

▭No

8. Does it take less than 15 min. to prepare the operating room?

▭Yes

▭No

9. Does it take less than 15 min. to take and have a CXR ready for the trauma team?

▭Yes

▭No

10. Does the hospital have an updated trauma protocol (a collection of procedures for the initial treatment of the injured patient)?

▭Yes

▭No

11. Does the hospital hold a customized registration form or checklists for trauma?

▭Yes

▭No

12. Does the hospital hold fixed criteria for transfer of trauma patients to the higher level of care?

▭Yes

▭No

13. Please state the number of patients transferred to hospitals with higher level of care each year?

_________ (Exact, which year)

_________ (Estimated)

14. Does the hospital hold a database or register for registration of trauma patients?

▭Yes

▭No

15. Does the hospital have regular trauma meetings auditing management and outcomes of trauma patients?

▭Yes (minimum twice per year)

▭No

16. If yes, how often?

Text: ______________

17. Regarding the senior surgeon present in the trauma team (or, if there are no trauma teams,the surgeon responsible for trauma care)

a. Is there a requirement that the surgeon should have partaken in the ATLS course?

▭Yes

▭No

b. Is there a requirement that the surgeon should have partaken in either the BEST course in haemostatic emergency surgery, War Surgery Course or the DSTC (Definitive Surgical Trauma Care) course?

▭Yes

▭No

18. Is the senior surgeon in the trauma team:

▭Available around the clock

▭Available to consult within 15 min

▭Available to consult within 30 min

19. Regarding the senior anesthesiologist in the trauma team team (or, if there are no trauma teams, the anaesthesiologist responsible for trauma care):

Is it required that the anesthesiologist should have partaken in the ATLS course?

▭Yes

▭No

20.Is the senior anaesthesiologist in the trauma team:

▭Available around the clock

▭Available to consult within 15 min

▭Available to consult within 30 min

21. Is there a requirement that at least one of the nurses in the trauma team has partaken in a trauma nursing course (TNCC course or equivalent)?

▭Yes

▭No

22. Does the hospital have around the clock emergency surgical function?

▭Yes

▭No

23. Does the hospital accept trauma patients around the clock?

▭Yes

▭No

24. What is the hospitals defined demographic area of coverage for trauma?

approx. _________ persons

22. How many trauma team activations took place in 2009 (if not 2009, then 2008).

___________ (Exact)

___________ (Estimated)
